# Supplementary material for: Recurrence Outcome in Hepatocellular Carcinoma within Milan Criteria Undergoing Microwave Ablation with or without Transarterial Chemoembolization
Source: Medicina (Kaunas). 2022 Jul 29;58(8):1016. doi: 10.3390/medicina58081016 (PMC9412845; doi:10.3390/medicina58081016)
Supplement: Supplementary file 1 [file medicina-58-01016-s001.zip › medicina-1808211-supplementary.pdf]

Supplementary Materials: Recurrence Outcome in Hepatocellular Carcinoma within Milan Criteria Undergoing Microwave Ablation with or without Transarterial Chemoembolization

Guobin Chen †, Hong Chen †, Xing Huang, Sisi Cheng, Susu Zheng, Yanfang Wu, Tanghui Zheng, Xiaochun Chen, Xinkun Guo, Zhenzhen Zhang, Xiaoying Xie* and Boheng Zhang*

Supplement Table S1. The analysis of mean LTPt and RFS around propensity score matching

Variable	pre-match			post-match		
	number	mLTPt(months)	mRFS(months)	number	mLTPt(months)	mRFS(months)
MWA	44	20.10	9.70	11	25.00	10.36
MWA+TACE	22	19.57	18.82	11	21.58	18.82
p Value		0.575	0.515		0.945	0.28

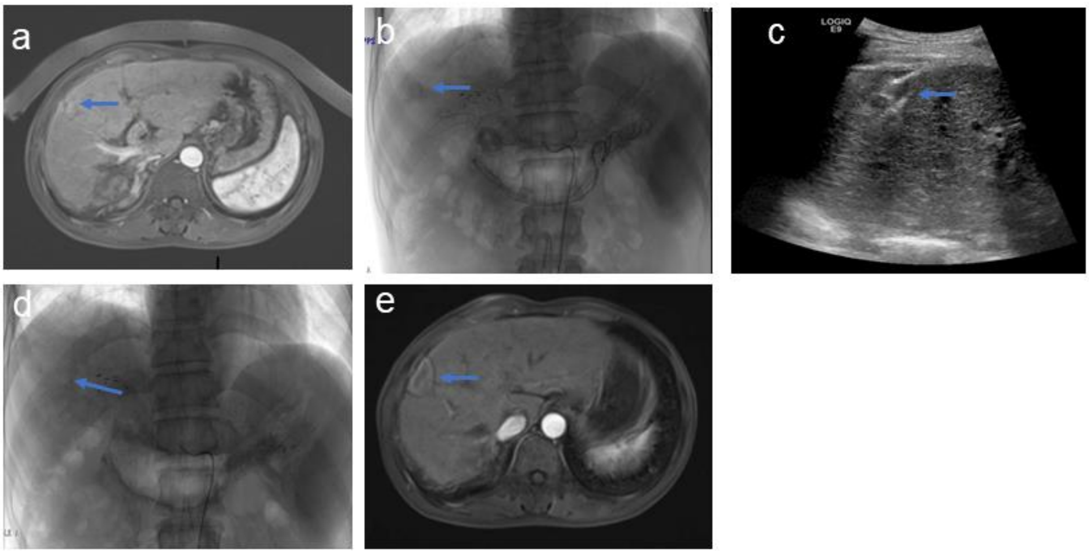

Supplement Figure S1. The procedures of TACE combined with MWA for a 54-years-old male with HCC. **a:** Preoperative CE-MRI found a tumor nodule in the subscapular of right lobe. **b:** Angiography demonstrated the location of the tumor and tumor-supplying arteries. **c:** The puncture was inserted through the tumor and performed the MWA. **d:** The embolization of tumor-feeding branch and angiography shown that tumor staining disappeared. **e** After 1 month of treatment, CE-MRI displayed tumor necrosis.
